# Impact of prenatal alcohol exposure on neurodevelopmental outcomes: a systematic review

**DOI:** 10.1080/21642850.2022.2129653

**Published:** 2022-10-06

**Authors:** Joanna Ting Wai Chu, Jessica McCormack, Samantha Marsh, Alesha Wells, Holly Wilson, Chris Bullen

**Affiliations:** a National Institute for Health Innovation, School of Population Health, University of Auckland, Auckland, New Zealand; b Social and Community Health, School of Population Health, University of Auckland, Auckland, New Zealand; c Department of Psychological Medicine, University of Auckland, Auckland, New Zealand

**Keywords:** Prenatal alcohol exposure, neurodevelopmental outcomes, pregnancy, alcohol, systematic review

## Abstract

**Background:**

Prenatal exposure to alcohol (PAE) represents a significant public health concern. Previous research linking PAE to neurodevelopmental outcomes has been mixed and often has limited focus on residual confounding or moderating factors.

**Methods:**

A systematic review of prospective cohort studies (*n* = >1000) assessing the impact of PAE on neurodevelopmental outcomes was undertaken (neurophysiology, motor skills, cognition, language, academic achievement, memory, attention, executive function, affect regulation, and adaptive behaviour, social skills, or communication). Electronic searches of EMBASE, Medline, CINAHL, and Psychinfo were conducted in May 2021. A quality assessment was conducted using an adapted version of the Newcastle-Ottawa Scale (NOS).

**Results:**

Thirty longitudinal cohort studies met the inclusion criteria. Evidence of the impact of PAE was mixed across domains. We found no evidence that PAE affects executive function, but there were impacts on motor skills, cognition, language, academic achievement, attention, affect regulation, and adaptive behaviour. The most consistent adverse effect was on affect regulation (nine out of thirteen studies, six of which found an association between heavy alcohol consumption or binge drinking during pregnancy). We found no protective factors. Few studies controlled for variables in the postnatal environment.

**Discussion:**

This review was unable to conclude a safe level of alcohol consumption during pregnancy. Methodological improvements are needed to improve the quality and consistency in which PAE is studied. Further research into residual confounding variables is vital, including a greater focus on the postpartum environment.

## Introduction

Guidelines for alcohol use in pregnancy advise that there is no known safe level of alcohol consumption during pregnancy and that it is safest to avoid alcohol if pregnant or planning to get pregnant (Carson et al., 2010; Carson et al., 2017; Department of Health and Social Care, 2016; National Health and Medical Research Council, 2009). Alcohol is a known teratogen that has a range of adverse effects on fetal development including major congenital abnormalities or functional defects to organs and changes in brain development associated with cognitive and behavioural changes in children (Carson et al., 2010; Ghazi Sherbaf et al., 2019; Gupta et al., 2016).

Fetal Alcohol Spectrum Disorder (FASD) is a diagnostic term that describes the neurological and physical effects of Prenatal Alcohol Exposure (PAE) (Harding et al., 2019). FASD is associated with a broad range of behavioural, cognitive, emotional, and adaptive functioning deficits, which can affect functional abilities and lead to adverse life outcomes in education, justice, and health (Streissguth et al., 2004). Without diagnosis individuals with FASD are unlikely to receive adequate or tailored care to match their needs, or may be misdiagnosed resulting in inadequate support (Skorka et al., 2020). In New Zealand, the Ministry of Health estimates that 1–3 in every 100 births may be affected by alcohol (Ministry of Health, 2020). Global prevalence of FASD is estimated at 7.7 per 1000 children (95% CI 4.9–11.7), however, the prevalence of FASD varies considerably by country, with high estimates in countries with pervasive drinking populations (e.g. South Africa: 111.1 per 1000 [95% CI, 71.1–158.4]; Ireland: 47.5 per 1000 [95% CI, 28.0–73.6]) (Lange et al., 2017).

Despite this, the rates of prenatal alcohol consumption remain high: global estimates suggest that 9.8% of pregnancies are exposed to alcohol (95% CI, 89–11.1%) (Popova et al., 2018). A New Zealand cohort study (*n* = 6822) found 23% of women reported drinking alcohol during pregnancy, 13% after the first trimester (Rossen et al., 2018). Australian research highlighted the high rates of alcohol intake between conception and recognition of pregnancy, with rates as high as 60.6%, decreasing to 18.3% after pregnancy recognition (McCormack et al., 2017). In the USA, surveys find that approximately one in eight pregnant women report alcohol use (500,000 per year), 80,000 of which reported binge drinking (Floyd & Sidhu, 2004). There is a great need for clear and consistent messaging to women regarding alcohol consumption, as well as adequate support for those moving to abstinence. These findings suggest that FASD and PAE is a significant public health concern in many Western countries. The impact of prenatal alcohol exposure is not limited to Western countries (Popova et al., 2018) and is likely extensive worldwide, for example among rural communities in Asian countries. The lack of data available on the effect of PAE on neurodevelopmental outcomes for these regions, likely due to FASD being largely unrecognised or misdiagnosed (Adnams, 2017), limits analysis of the degree of impact.

While evidence shows that alcohol is generally harmful to the fetus, the impact of PAE is subject to individual and environmental factors, such as diet, genetics, maternal stress, tobacco smoking, use of marijuana and other substances, as well as the postnatal environment (Australian Government National Health Medical Research Council, 2009; Jacobson et al., 2004; Murphy et al., 2013; National Health and Medical Research Council, 2009). Previous studies suggest that heavy PAE is adversely associated with a number of neurocognitive domains, including behaviour, affect regulation and cognition (Khoury et al., 2015). However, findings are mixed with respect to low and moderate levels of alcohol use.

Recent systematic reviews of the literature consistently fail to provide any definitive conclusions regarding the effects of low to moderate alcohol consumption. For example, a recent systematic review of the impact of alcohol consumption on ADHD-like symptoms found that low alcohol consumption (up to 50 g) decreased the odds of ADHD-like symptoms in male offspring, with no effect of PAE on the development of ADHD-like symptoms observed for male or female offspring (San Martin Porter et al., 2019). This review only included studies controlling for key confounds, including maternal smoking during pregnancy, SES, age, and maternal education, with minimal discussion or reporting of additional confounds. An additional review published the same year examined the impact of low to moderate alcohol consumption more broadly on mental health problems of offspring (Easey et al., 2019). The results suggested a negative link between alcohol consumption and offspring mental health. However, analysis and conclusions were limited by the quality of included studies, some with low samples sizes and potentially underpowered, others utilising a categorical measure of alcohol exposure (yes/no). Finally, a systematic review of evidence examining the link between low alcohol consumption and pregnancy and childhood outcomes aimed to reduce biases by only including those with prospective measure of alcohol consumption and prioritising those who controlled for key confounds (SES, maternal smoking during pregnancy, maternal age, and ethnicity) (Mamluk et al., 2017). With results indicating some support for the negative impact of low alcohol consumption on preterm birth and birth weight, with mixed or null findings for other outcomes such as IQ and academic achievement.

Given the role of environmental factors and the inconsistencies in the relationship between PAE and neurodevelopmental outcomes, perhaps there are important environmental and maternal factors that moderate the impact of PAE on neurodevelopmental outcomes, such as the quality of the postnatal environment and ongoing alcohol use. Further research is needed to understand the impact of PAE on neurodevelopmental outcomes and the variables that moderate or exacerbate the effect of alcohol on long-term outcomes. This study, therefore, aims to detect the effect of PAE on neurodevelopmental outcomes and investigate any confounding variables that may moderate this relationship. To achieve this a comprehensive review of large cohort-based studies, as large studies (>1000 participants) are sufficiently powered to detect the impact of different levels of alcohol consumption, across all neurodevelopmental domains was conducted. This review differentiates itself by including a narrative review of confounding factors included in each study's statistical analyses to further understand how environmental or maternal factors moderate the impact of PAE on offspring and by including large cohort-based studies which have the ability to detect the effect of PAE on outcomes.

## Methods

A systematic review was conducted according to the Preferred Reporting Items for Systematic Reviews and Meta-Analyses (PRISMA) (David Moher et al., 2009). The protocol was registered in PROSPERO 2021 CRD42021256407. An ethics statement is not applicable because this study is based exclusively on published literature.

### Search strategy

We conducted electronic searches of the following databases: EMBASE, Medline, CINAHL, and Psychinfo. Search terms included alcohol (and consum* or expos* or drink*) and (matern* or pregnan* or f?etal or prenatal), combined with keywords for the outcomes (e.g. executive function, motor movement, language) and design (e.g. prospective, birth cohort). Searches were limited to peer reviewed English language studies of human participants published after January 2001, to ensure that the review only incorporated recent research. An example of the search strategy can be found in Appendix 1. All searches were conducted on 25th of May 2021 and exported to Endnote. Additional articles were identified from the bibliographies of relevant systematic reviews.

### Inclusion and exclusion criteria

The inclusion and exclusion criteria are summarised in Table 1. Studies were included if they were prospective cohort studies assessing neurodevelopmental outcomes in children exposed to alcohol in utero compared to unexposed children. Relevant outcomes included neurodevelopmental or neurocognitive outcomes associated with FASD including the 10 domains identified in the Canadian Guidelines for diagnosing FASD: neurophysiology, motor skills, cognition, language, academic achievement, memory, attention, executive function, affect regulation, and adaptive behaviour, social skills, or communication (Cook et al., 2016). Retrospective and case-control studies were excluded due to a high risk of recall bias. Due to the relatively low prevalence of alcohol use during pregnancy, a minimum of 1000 participants were required in each study to have sufficient data for analysis, two studies (Donald et al., 2019; Halliday et al., 2017) included in the analysis had less than 1000 participants in the final analysis, but originally recruited populations larger than 1000 (1143 and 1038, respectively) so were included in this systematic review. Studies were required to include a quantitative measure of alcohol consumption (e.g. standard drinks, grams of alcohol).

**Table 1.** Inclusion/exclusion criteria.

	Inclusion	Exclusion
Population	Pregnant women sampled from the population and their offspring (under the age of 18 years at time of outcome assessment)	Postnatal women Adult off-spring
Exposure	Any level of prenatal alcohol consumption. Must include a quantitative measure of alcohol consumption	Alcohol must be the main exposure, or if multiple exposures, it must be an independently evaluated variable
Comparator	Women that did not consume alcohol in pregnancy.	Studies without a comparator will be excluded
Outcome	Neurodevelopmental outcomes related to FASD: e.g. developmental delay; motor skills/function; neurophysiology; cognition; cognitive development; language; academic achievement; IQ; memory; attention; executive function; affect regulation; behaviour complications; adaptive behaviour; social skills; communication	Studies of unrelated outcomes will be excluded
Study Design	Prospective cohort studies	Retrospective and case-control studies. *N* > 1000

### Study selection and data extraction

Titles and abstracts were screened by the reviewer (AW) to identify relevant studies. The full text of potentially relevant articles was obtained to determine their inclusion. A second reviewer (JM) independently screened a random selection (10%) of articles, and any discrepancies were discussed, and disagreements were resolved by consensus. Data were extracted using a previously designed extraction form and included the following variables: design, location, population, exposure (timing, amount), measurement method, moderators included in the model, effect of moderators, outcomes and method of measurement, and results. Data were extracted by the first reviewer and checked for accuracy by the second reviewer.

### Quality

A quality assessment was conducted by both reviewers of the included studies using an adapted version of the Newcastle-Ottawa Scale (NOS) (Penson et al., 2012). This scale has been used in similar previous research (Sundermann et al., 2019). It contains eight questions and scores from 0 (high risk of bias) to 9 (low risk of bias). Questions address representativeness of the cohort, measurement of exposure, statistical analyses (control for confounds), assessment of outcomes, and characteristics of the follow-up.

### Analysis

Study information was summarised into a table and a narrative review of the literature was conducted to summarise the findings across the studies and to explore any effects of confounding factors. A meta-analysis was planned, however, due the heterogeneity in studies, across how and when PAE was measured, variation in neurodevelopmental domains investigated, how they were measured and age of offspring, a meta-analysis would not have been useful to summarise the findings, consistent with previous reviews in this area (Easey et al., 2019; Mamluk et al., 2017). A narrative review was preferred as this approach enables common patterns to be identified across studies with methodological differences. The aims of this research were to understand the impact of PAE on child development and to identify important moderating variables of the effects of PAE. Results are therefore summarised by neurodevelopmental outcome and a final examination of common moderators included in this research and their effects.

## Results

**Figure 1.**
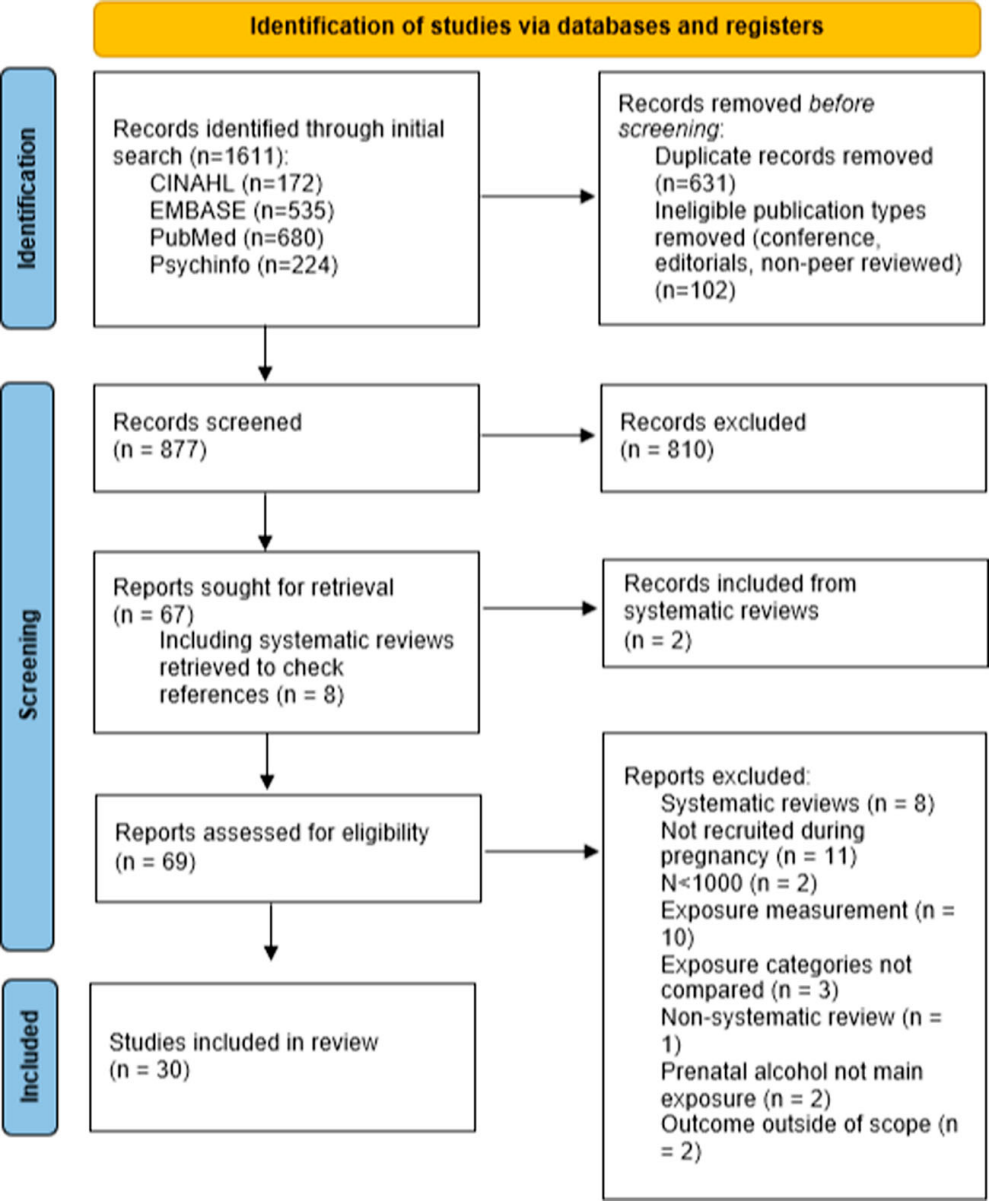
PRISMA flow chart of included studies.

The database searches resulted in 1611 articles (see Figure 1). Of these 631 were identified as duplicates and 102 were identified as ineligible publication types. The remaining 877 records were screened via title and abstract, excluding 810 as irrelevant. Full-text review of 67 articles, including 8 systematic reviews (reference lists checked) which produced 2 additional relevant studies. Of these, 30 studies were included in the review. Twelve were from Denmark, seven were from the UK, five were from Australia, two each from Norway and New Zealand, and one each from Brazil and South Africa. The birth cohorts were recruited between 1981 and 2015.

Most studies interviewed women about their alcohol use around 16–20 weeks gestation (*n* = 24). Self-reported retrospective recall was used in all studies. Alcohol use was reported for multiple time periods: pre-pregnancy (*n* = 2); early pregnancy (*n* = 7); first trimester (*n* = 18); second trimester (*n* = 3); third trimester (*n* = 7); any time in pregnancy (*n* = 6). In the UK one standard drink is classified as containing 8 g of alcohol, whereas Australia and NZ quantify a standard drink to contain 10 g of alcohol, and 12 g in Scandinavian countries. Binge drinking was generally classified as having greater than four or five drinks per occasion.

Across the 10 domains identified by Cook et al. (2016) the majority of the included studies explored affect regulation (43.3%), cognition (36.7%), and attention (26.7%). Most studies looked at multiple domains. No studies meeting criteria evaluated memory or neurophysiology. An overview of study characteristics is provided in Table 2 and each studies characteristics is provided in Table 3. Under the new Canadian FASD diagnostic guidelines hyperactivity and inattention fall under separate categories (executive function and attention respectively) (Cook et al., 2016), however, we have classified scales that combine hyperactivity and inattention as attention only. Outcomes were assessed in offspring between 6 months and 19 years, one study (Weile et al., 2020) included participants up to the age of 19, this study was included in the analysis as it had a large sample size and all of the rest of studies included examined participants below the age of 18 . The majority of studies evaluated outcomes assessed in children under six years of age (*n* = 19). Most studies included in our analysis were deemed high quality, scoring between 7 and 9 (66.7%) and none of the studies were deemed very high risk, scored on the NOS. Results of quality assessment can be found in Appendix 2.

**Table 2.** Summary of study outcome characteristics.

Outcome assessed	*N*	%
*Neurodevelopmental domain assessed*		
Neurophysiology	0	0
Motor Skills	5	16.7
Cognition	11	36.7
Language	3	10.0
Academic achievement	5	16.7
Memory	0	0
Attention	8	26.7
Executive function	3	10.0
Affect regulation	13	43.3
Adaptive behaviour, social skills, or communication	2	6.7
*Age at outcome assessment*		
<2 years of age	9	30.0
3–5 years of age	12	40.0
6–12 years of age	9	30.0
13–18 years of age	3	10.0
Up to 19 years of age	1	3.3

Note*:* Studies reported on multiple outcomes, therefore the percentages displayed above do not add to 100.

**Table 3.** Summary of each study included in the analysis.

Author, year	Country	Number of (offspring) participants in analysis	Neurophysiological domain(s) investigated	Outcome(s) measure or scale	Timing of PAE	Offspring age at assessment	Association between PAE and neurophysiological domain investigated
Alati et al., 2013	UK	7062	Academic Achievement	Key Stage 2 (KS2)	Alcohol consumption during first trimester, measured at 18 weeks of gestation	11 years	Academic achievement: No association with daily drinking, but association between consumption of four or more drinks per occasion and lower academic abilities
Alati et al., 2008	UK	4332	Cognition	IQ – WISC-III	Alcohol consumption during first trimester, measured at 18 weeks of gestation	8 years	Cognition: no significant association
Alvik et al., 2013	Norway	1138	Affect regulation	SDQ	Alcohol consumption during 0–6 weeks of pregnancy, measured at 17 weeks	5.5 years	Affect regulation: binge drinking during the early stages of pregnancy predicted scores in the Abnormal and Borderline range of the SDQ among the cohort of 5.5-year-olds. A binge drinking rate of less than once a week was associated with higher Total Problem scores on the SDQ, and greater than or equal to once a week predicted elevated risk of abnormal or borderline scores for total problems, emotional subscale, and conduct subscale
Alvik et al., 2011	Norway	1330	Affect regulation	Infant Characteristics Questionnaire:temperament	Alcohol consumption during 0–6, 7–12 and 13 + weeks of pregnancy, measured at 17 weeks	6 months	Affect regulation: Binge drinking more than once a week during 0–6weeks is associated with a difficult temperament
D’Souza et al., 2019	New Zealand	5768	Language, Affect Regulation	Comorbidity of language and behavioural difficulties (MB-CDI and SDQ)	Assessed in the 3rd trimester covering the 1st trimester.	2 years	Language: consumption of one or more drinks per week in the first trimester was associated with reduced likelihood of language difficulties, relative to those who abstained. Affect regulation: no significant association
Donald et al., 2019	South Africa	734	Motor skills, Cognition, Language	BSID-III subscale	Recruited at 28–32 weeks gestation, alcohol consumption assessed covering the whole pregnancy	2 years	Motor skills: No significant association Cognition: No significant association Language: significant positive association with receptive language deficits
Faebo Larsen et al., 2013	Denmark	33,354	Motor skills	DCDQ’07	16 weeks gestation covering the first trimester	7 years	Motor skills: No association between PAE and developmental co-ordination disorder
Falgreen Eriksen et al., 2012	Denmark	1628	Cognition	IQ (WPPSI-R)	17 weeks gestation covering ‘early pregnancy’	5 years	Cognition: PAE of greater than or equal to nine drinks per week in early pregnancy increased risk of low full-scale and verbal IQ. No differences were observed when mothers reported consuming 1–4 standard drinks or 5–8 standard drinks per week relative to those who abstained
Halliday et al., 2017	Australia	554	Motor Skills, cognition, language, affect regulation, (adaptive behaviour, social skills or communication)	BSID-III subscale Brief Infant Toddler Social Emotional Assessment – Competence scale	19, 26 weeks gestation, following birth. Exposure categories Trimester 1 (pre-pregnancy awareness), Trimester 2 (post-awareness), Trimester 2 and Trimester 3	2 years	Motor skills: No significant association Cognition: protective effect of low levels of drinking on improved cognition, but this difference was accounted for by moderating variables Language: no association with language deficits Affect regulation: found that early binge drinking with continued low level alcohol consumption over the duration of the pregnancy was associated with lower sensation avoidance scores in offspring at age 2 Adaptive behaviour, social skills or communication: no significant associations with social competencies
Hutchinson et al., 2019	Australia	1324	Motor skills, cognition	BISD-III	Trimester one (weeks 0–6), trimester one (weeks 7-12), trimester two, trimester three	12 months	Motor skills: no significant association Cognition: no significant association
Kesmodel, Bertrand, et al., 2012	Denmark	1628	Cognition, Attention, Executive function	WPPSI-R, TEACh-5, BRIEF	17 weeks gestation covering early to mid-pregnancy	5 years	Cognition: no significant association Attention: no significant association Executive function: no significant association
Kesmodel, Eriksen, et al., 2012	Denmark	1617	Cognition	WPPSI-R	17 weeks gestation covering early to mid-pregnancy	5 years	Cognition: no significant difference in IQ between the children of mothers who reported binge drinking and mothers with no binge episodes, however, they did find that binge drinking during gestational weeks 1–2 was associated with reduced risk of low full-scale IQ compared to those unexposed to binge drinking
Kilburn et al., 2015	Denmark	1628	Executive Function	CRT and Information processing time	17 weeks gestation covering early pregnancy	60–64 months	Executive function: no significant effect on reaction time or information processing
McCormack et al., 2018	Australia	1331	Cognition	BSID-III	Trimester one (weeks 0–6), trimester one (weeks 7–12), trimester two, trimester three	12 months	Cognition: low level consumption of alcohol during the second and third trimester was associated with slightly higher cognitive scores, relative to mothers who abstained
Negrao et al., 2020	Brazil	1006	Motor skills, Cognition	BSID-III subscale	A few days after birth of the child covering the whole pregnancy	2 years	Motor: found an association between concomitant use of alcohol and tobacco and fine motor skills, but not for gross motor skill or for alcohol consumption alone. Children whose mothers’ used alcohol and tobacco concomitantly during pregnancy were at increased risk for motor delays in fine motor skills, but not in gross motor delay Cognition: no significant association
Niclasen, Andersen, et al., 2014	Denmark	37,315	Affect Regulation	SDQ	Weeks 16 and 30 gestation, and 6 months postpartum.	7 years	Affect regulation: association between affect regulation and exposure to binge episodes only in male children. Exposure to binge episodes was associated with an increased change in mean for externalising behaviours compared to children whose mothers reported no binge episodes and internalising behaviours – although the effect was not significant at four or more binge episodes
Niclasen, Nybo Andersen, et al., 2014 (Teasdale)	Denmark	37,315	Attention, Affect Regulation, (Adaptive behaviour, social skills, or communication)	SDQ subscale(s)	Weeks 16 and 30 gestation, and 6 months postpartum.	7 years	Attention: no association with hyperactivity Affect regulation: significant association between binge drinking and externalising scores in early pregnancy and late pregnancy Adaptive behaviour, social skills or communication: there was a significant increased risk of abnormal or borderline scores on the peer problems scale for children not exposed to alcohol compared to children to a cumulative 15–45 drinks over the pregnancy), no association for other amounts of alcohol exposure
O’Callaghan et al., 2007	Australia	5139	Cognition, Academic achievement, attention	Raven progressive matrices WART-R; learning difficulties CBCL subscale	20 weeks gestation, days after delivery covering early and late pregnancy	14 years	Cognition: significant association between binge drinking and IQ. No significant association between alcohol exposure in early pregnancy and IQ, however, there was a higher prevalence of children with poor Raven's scores in children exposed to binge drinking and children exposed to alcohol in late pregnancy. The adjusted model found that there was an increased risk for low IQ for children whose mother reported engaging in binge drinking compared to those that reported no binge episodes Academic achievement: no association with low scores on the WRAT-R Attention: no significant association
Robinson et al., 2010	Australia	1860	Affect Regulation	CBCL	Assessed 18 and 34 weeks – covering the whole pregnancy	2, 5, 8, 10 and 14 years	Affect regulation: light to moderate levels of drinking in early pregnancy was associated with a reduced likelihood of scores above the clinical cut-off on the CBCL in children aged between 2 and 14 years compared to unexposed children on the externalising, internalising, and total behaviour
Rodriguez et al., 2009	Denmark, Finland	21,678	Attention	SDQ subscale or Rutter Scale subscale	Between 12- and 32-weeks gestation covering the whole pregnancy	15 years	Attention: no significant association with hyperactivity/inattention
Sayal et al., 2013	UK	10,558	Academic Achievement, Affect regulation	KS2	18 weeks gestation covering the first trimester	11 years	Academic achievement: no significant associations Affect Regulation: no significant associations
Sayal et al., 2014	UK	7965	Academic Achievement, Attention, Affect regulation	KS2 SDQ subscale	18- and 32-weeks gestation covering the first trimester	10–11 years	Academic achievement: drinking 4 + drinks per occasion was significantly associated with lower KS2 scores Attention: significant association for girls at age 11 on the parent rated SDQ hyperactivity/inattention subscale and binge drinking. Further analyses separating the effects of daily drinking and binge drinking found significant effects for the exposure category 4 + drinks but not daily drinking and the parent rated SDQ subscale for girls and the teacher rated SDQ subscale for the whole sample. Affect regulation: binge pattern of alcohol consumption was associated with elevated conduct problem scores among girls (age 11) compared to the children of mothers who reported no binge episodes, as well for total problems. There was no significant association in male children or for daily non-binge drinking patterns.
Sayal et al., 2009	UK	8240	Cognition Attention Affect regulation	WPPSI-R SDQ	18- and 32- weeks gestation covering the first trimester	47 months	Cognition: no association with IQ score Attention: 4 plus drinks a day were significantly associated with higher scores (more problematic) on the hyperactivity/inattention subscale of the SDQ. Affect regulation: binge patterns of alcohol consumption during the second and third trimester was associated with poorer scores on both the conduct and total problems scale of the SDQ among girls at 47 months compared to children not exposed to binge drinking. Elevated scores on the conduct problems subscale and total problems were apparent across the whole sample during the 81-month data collection window.
Sayal et al., 2007	UK	47 months (*N* = 9086), 81 months (*N* = 8046), 93 and 108 months (*N* = 5648)	Affect regulation	SDQ	18 weeks gestation covering the first trimester	47, 81, 93, 108 months	Affect Regulation: alcohol intake at less than one drink per week was associated with poor total problems scores at 47 months, 81 months, and 93-108 months among girls. Teacher rated conduct score were also elevated in this exposure category for the whole sample at 93–108 months. However, the association between PAE and total problems scores was not significant for one or more drinks per week or for male children.
Schoeps et al., 2018	New Zealand	6156	Affect regulation	Infant Behaviour Questionnaire (IBQ-R VSF); SDQ	Last trimester covering the first and last trimester	9 months and 2 years	Affect regulation: four or more drinks per week was associated with children with higher total difficulties.
Skogerbo et al., 2013	Denmark	1628	Affect Regulation	SDQ	17 weeks covering early pregnancy	5 years	Affect Regulation: no significant associations
Skogerbo et al., 2012	Denmark	1628	Executive function	Parent rated BRIEF	17 weeks covering early pregnancy	5 years	Executive function: significant associations between binge drinking in gestational week 9 or later and elevated parent ratings on the behaviour regulation index and teacher rated metacognition index
Underbjerg et al., 2012	Denmark	1628	Attention	TEACh-5	17 weeks covering early pregnancy	5 years	Attention: significant association between consumption of 9 or more drinks per week and low overall attention score, no associations between binge drinking and attention
Weile et al., 2020	Denmark	48,072	Attention	ADHD diagnosis	11 weeks gestation covering early pregnancy	Up to 19 years of age	Attention: no significant associations with ADHD diagnosis
Zuccolo et al., 2013	UK	4061 – 8 years 6268 – 11 years	Cognition, academic achievement	WISC, KS2	18 weeks gestation covering the first trimester	Age 8 and 11	Cognition: no significant association Academic achievement: no significant association

### Motor skills

Five of the included studies examined the impact of prenatal alcohol exposure on motor skills (Donald et al., 2019; Faebo Larsen et al., 2013; Halliday et al., 2017; Hutchinson et al., 2019; Negrao et al., 2020). Four of the studies assessed motor skills during infancy using the Bayley Scales of Infant and Toddler Development (BSID-III; Bayley (2006)), a validated tool for assessing development conducted by trained professionals. Three of the four studies found no significant association between PAE and motor skills (Donald et al., 2019; Halliday et al., 2017; Hutchinson et al., 2019). Negrao et al. (2020) found an association between concomitant use of alcohol and tobacco and fine motor skills, but not for gross motor skill or for alcohol consumption alone. Compared to children whose mother did not use alcohol or tobacco, children whose mothers’ used alcohol and tobacco concomitantly during pregnancy were at increased risk for motor delays in fine motor skills *(RR* 2.81, 95% CI [1.65, 4.77], *p *< 0.05), but not in gross motor delay (*RR* 1.49, 95% CI [0.77, 2.87], *p *> 0.05).

Only one study assessed motor skills after 2 years; in Faebo Larsen et al. (2013) motor skills were assessed at age seven using the Developmental Coordination Disorder Questionnaire 2007 (Wilson et al., 2009), which is a parent self-report measure. There was no association between PAE and developmental co-ordination disorder at 7 years.

### Cognition

A total of 11 studies used cognition as an outcome measure (Alati et al., 2008; Donald et al., 2019; Falgreen Eriksen et al., 2012; Halliday et al., 2017; Hutchinson et al., 2019; Kesmodel, Bertrand, et al., 2012; Kesmodel, Eriksen, et al., 2012; McCormack et al., 2018; O’Callaghan et al., 2007; Sayal et al., 2009; Zuccolo et al., 2013). Seven of the studies evaluated the impact of PAE on IQ: four studies evaluated IQ in children 5 years or younger (Falgreen Eriksen et al., 2012; Kesmodel, Bertrand, et al., 2012; Kesmodel, Eriksen, et al., 2012; Sayal et al., 2009) using the Wechsler Preschool and Primary Scale of Intelligence (Wechsler, 2012); two studies reporting to the ALSPAC cohort assessed IQ in 8-year-olds IQ (Alati et al., 2008; Zuccolo et al., 2013) using the Wechsler Intelligence Scale for Children (Wechsler, 2003); and one evaluated IQ in teenagers (O’Callaghan et al., 2007) using the Raven Progressive Matrices (Raven et al., 1998). Only three of the studies found a significant association between PAE and IQ, two of which drew on data from the Danish National Birth Cohort at 5 years. Falgreen Eriksen et al. (2012) reported that there was an increased risk of low full-scale (OR 4.6; 95% CI [1.2, 18.2]) and verbal IQ (OR 2.9, 95% CI [1.4, 24.9]) when exposed to greater than or equal to 9 drinks per week in early pregnancy compared to unexposed children. No differences were observed between children whose mothers reported consuming 1–4 standard drinks or 5–8 standard drinks per week compared to children of mothers who reported abstaining from alcohol. Kesmodel, Eriksen, et al. (2012) found no significant difference in IQ between the children of mothers who reported binge drinking and mothers with no binge episodes, however, they did find that binge drinking during gestational weeks 1–2 was associated with reduced risk of low full-scale IQ compared to those unexposed to binge drinking (*OR* 0.54; 95% CI [0.31, 0.96], *p *< 0.05). This latter finding may be explained by unexplored confounding variables as no other exposure timings were significant.

Finally, O’Callaghan et al. (2007) found a significant association between binge drinking and IQ scores as measured by Raven Progressive Matrices. There was no significant association between alcohol exposure in early pregnancy and IQ, however, there was a higher prevalence of children with poor Raven's scores (i.e. <85) in children exposed to binge drinking and children exposed to alcohol in late pregnancy. The adjusted model found that there was an increased risk for low IQ for children whose mother reported engaging in binge drinking compared to those that reported no binge episodes (OR 1.4, 95% CI [1.1, 1.8], *p *< 0.05).

The four remaining studies evaluated cognition using the cognition subscale of the BSDI-III (Donald et al., 2019; Halliday et al., 2017; Hutchinson et al., 2019; McCormack et al., 2018). Only one study using the BSID-III to assess cognition found any significant differences between exposure groups. McCormack et al. (2018) assessed cognition in infants at 12 months of age and found that compared to mothers who abstained, low-level consumption of alcohol during the second and third trimester was associated with slightly higher cognitive scores (Second trimester: β = 2.11, SE 0.77, *p *< 0.01; Third trimester: β = 1.60, SE 0.77, *p *< 0.05). There were no significant differences between mothers who abstained and the different levels of alcohol consumption for the first trimester.

### Language

Across the 30 studies included in this review, only three studies examined the impact of prenatal alcohol exposure on language abilities (Donald et al., 2019; D’Souza et al., 2019; Halliday et al., 2017). All three studies assessed at two years using either a subscale of the BSID-III or the MacArthur-Bates Communicative Development inventory (Hutchins, 2013). D’Souza et al. (2019) found that compared to those who abstained, consumption of one or more drinks per week in the first trimester was associated with reduced likelihood of language difficulties (OR 0.61, 95% CI [0.48, 0.79]). However, the study noted that there were a low number of individuals in the sample reporting moderate or severe drinking. Similarly, Donald et al. (2019) found a significant positive association between alcohol use during pregnancy and receptive language deficits (β = 0.57, 95% CI [0.33, 0.96], *p *< 0.05). Halliday et al. (2017) found no association between PAE and language deficits.

### Academic achievement

Five studies assessed academic achievement outcomes (Alati et al., 2013; O’Callaghan et al., 2007; Sayal et al., 2013; Sayal et al., 2014; Zuccolo et al., 2013). Four of the studies used the Key Stage 2 (KS2), which is part of UK testing as part of the National Curriculum at age 11; all four were analyses of the ALSPAC cohort (Alati et al., 2013; Sayal et al., 2013; Sayal et al., 2014; Zuccolo et al., 2013). Two of the studies found an association between academic achievement and consumption of four or more drinks per occasion during pregnancy, although no association between daily drinking or lower levels of alcohol consumption (Alati et al., 2013; Sayal et al., 2014). Alati et al. (2013) found an increased risk of lower KS2 scores associated with frequent consumption of four or more units per occasion compared to children of mothers that never consumed alcohol (mean change 20.68, 95% CI [20.33, 21.03]). Likewise, Sayal et al. (2014) found that drinking 4 + drinks per occasion (but not daily drinking) was significantly associated with lower KS2 scores compared to women who reported no binge episodes during pregnancy (MD −0.81, 95% CI [−0.16, −1.46], *p *< 0.05). No significant association between PAE and academic achievement was found for low levels of alcohol consumption (Sayal et al., 2013) or non-binge alcohol use (Zuccolo et al., 2013).

The final study utilised the reading subscale of the Wide Range Achievement Test – Revised (WRAT-R; Jastak (1984)), administered to offspring at age 14, which found no association between PAE and low scores on the WRAT-R (O’Callaghan et al., 2007).

### Attention

Eight studies examined attention related outcomes (Kesmodel, Bertrand, et al., 2012; Niclasen, Nybo Andersen, et al., 2014; O’Callaghan et al., 2007; Rodriguez et al., 2009; Sayal et al., 2009; Sayal et al., 2014; Underbjerg et al., 2012; Weile et al., 2020). Two of the studies used a measure specifically for attention, that is, the Test Everyday Attention for Children at Five (TEACh-5:Manly et al. (2001)) (Kesmodel, Bertrand, et al., 2012; Underbjerg et al., 2012). Five of the remaining studies analysed the hyperactivity/inattention subscales from the Strengths and Difficulties Questionnaire (SDQ), Rutter Scale, or Child Behaviour Checklist (CBCL) (Niclasen, Nybo Andersen, et al., 2014; O’Callaghan et al., 2007; Rodriguez et al., 2009; Sayal et al., 2009; Sayal et al., 2014). One study used administrative data linking children in the birth cohort to subsequent diagnoses of ADHD up to age 19 (Weile et al., 2020).

Of the two studies that measured attention specifically, only one found an association between PAE and attention. Underbjerg et al. (2012) administered the TEACh-5 to a sample of 5-year-olds in Denmark finding a significant association between maternal consumption of nie or more drinks per week and a risk of a low overall attention score (OR 3.50, 95% CI [1.15, 10.68]). There were no significant associations observed between binge drinking and attention. This was similarly found in a separate analysis of the same cohort by Kesmodel, Bertrand, et al. (2012); after adjusting for a range of confounding variables there was no significant associated between frequency of alcohol consumption or presence of binge drinking and attention.

Only two studies found an association between PAE and hyperactivity/attention subscale scores. Sayal et al. (2014) found a significant association for girls at age 11 on the parent-rated SDQ hyperactivity/inattention subscale and binge drinking (mean difference 0.25, 95% CI [0.04, 0.47], *p *= 0.022). Further analyses separating the effects of daily drinking and binge drinking found significant effects for the exposure category 4+ drinks but not daily drinking and the parent-rated SDQ subscale for girls (mean difference 0.30, 95% CI [0.02, 0.58], *p *= 0.035) and the teacher-rated SDQ subscale for the whole sample (mean difference 0.28, 95% CI [0.04, 0.51], *p *= 0.024). These findings were consistent with an earlier analysis of the same cohort (ALSPAC), where the SDQ was administered at 47 and 81 months of age (Sayal et al., 2009). Episodes of 4 plus drinks a day were significantly associated with higher scores (more problematic) on the hyperactivity/inattention subscale of the SDQ at both time points (mean difference 0.32, 95% CI [0.03, 0.67], *p *= 0.032). However, there was no association between low levels of alcohol use and hyperactivity/inattention in the ALSPAC cohort at the same age (Sayal et al., 2007).

The final study utilised record linkage to obtain ADHD diagnoses from the Danish Psychiatric Central Research Register (Weile et al., 2020). The study found no significant associations between low levels of maternal alcohol consumption or binge drinking episodes in early pregnancy and rates of ADHD in offspring.

### Executive function

Three of the included studies analysed executive function: two of the studies used the Behaviour Rating Inventory of Executive Functions (BRIEF: Gioia et al. (2000)) (Kesmodel, Bertrand, et al., 2012; Skogerbo et al., 2012), and the third study assessed choice reaction time and information processing (Kilburn et al., 2015). All three studies assessed executive function in children aged 5 years. Kesmodel, Bertrand, et al. (2012) found no significant association between any level of maternal alcohol consumption and parent-rated BRIEF scores. However, research conducted by Skogerbo et al. (2012) found significant associations between binge drinking in gestational week 9 or later and elevated parent ratings on the behaviour regulation index (OR 2.04, 95% CI [0.33, 3.76]) and teacher-rated metacognition index (OR 2.06, 95% CI [1.01, 4.23]) among a cohort of 5-year-olds.

In Kilburn et al. (2015) reaction time and information processing were assessed using the Sternberg paradigm. The study found no significant effect of PAE on reaction time or information processing time across the different levels of weekly intake, or for binge drinking.

### Affect regulation

The association between PAE and affect regulation was evaluated in 13 studies. The majority of the studies (*n* = 9) analysed affect regulation in children between 5 and 11 years using the SDQ Total problems measure or the externalising and internalising subscales. Three of the studies evaluated affect regulation in infants using different measures (Alvik et al., 2011; Halliday et al., 2017; Schoeps et al., 2018). One study evaluated affect regulation using the CBCL (Robinson et al., 2010).

Eight studies found an association between PAE and affect regulation, with seven findings finding that PAE adverse affected emotional regulation. Niclasen, Andersen, et al. (2014) found a significant association between binge drinking and externalising scores in both early pregnancy (change in mean [RCM] 1.02, 95% CI [1.00,1.05]) and late pregnancy (RCM 1.21, 95% CI [1.04, 1.42]). Similarly, Niclasen, Nybo Andersen, et al. (2014) found an association between affect regulation and exposure to binge episodes for the same cohort, but only in male children. Exposure to binge episodes was associated with an increased change in mean for externalising behaviours compared to children whose mothers reported no binge episodes (1 binge episode: RCM 1.04, 95% CI [1.01, 1.07]; 2–3 binge episodes: RCM 1.07, 95% CI [1.04, 1.11]; 4 + binge episodes: RCM 1.01, 95% [0.94, 1.09], *p *> 0.05) and internalising behaviours (1 binge episode: RCM 1.04. 95% CI [1.00, 1.07]; 2–3 binge episodes: RCM 1.04, 95% [1.00, 1.08]; 4 + binge episodes: RCM 1.06, 95% CI [0.98, 1.15], *p *> 0.05) – although the effect was not significant at 4 or more binge episodes. There was no significant association between alcohol exposure and affect in girls, although there was a tendency towards increased relative mean change. Niclasen, Nybo Andersen, et al. (2014) found a small positive association between binge drinking and conduct scores in boys (1 binge episode: RCM 1.14, 95% CI [1.03, 1.27]; 2–3 binge episodes: RCM 1.23, 95% CI [1.08, 1.40]; 4 + binge episodes: 1.12, 95% CI [0.86, 1.45], *p *> 0.05).

Alvik et al. (2013) reported that binge drinking during the early stages of pregnancy predicted scores in the Abnormal and Borderline range of the SDQ among the cohort of 5.5-year-olds. A binge drinking rate of less than once a week was associated with higher Total Problem scores on the SDQ (OR 1.5, 95% CI [1.0, 2.1], *p *= 0.05), and greater than or equal to once a week predicted elevated risk of abnormal or borderline scores for total problems (OR 4.1, 95% CI [1.7, 9.8], *p *< 0.01), emotional subscale (OR 3.2, 95% CI [1.3, 8.0], *p *< 0.05), and conduct subscale (OR 3.0, 95% CI [1.3, 7.2], *p *< 0.05) compared to the children whose mothers reported no binge episodes.

Sayal et al. (2014) found that a binge pattern of alcohol consumption was associated with elevated conduct problem scores among girls (age 11) compared to the children of mothers who reported no binge episodes (adjusted MD 0.16, 95% CI [0.01, 0.31], *p *= 0.34), as well for total problems (adjusted MD 0.80, 95% CI [0.29, 1.31], *p *= 0.002). There was no significant association in male children or for daily non-binge drinking patterns. In an earlier wave of the same cohort, Sayal et al. (2009) found binge patterns of alcohol consumption during the second and third trimester was associated with poorer scores on both the conduct and total problems scale of the SDQ among girls at 47 months compared to children not exposed to binge drinking (conduct problems: adjusted MD 0.13, 95% CI [0.00, 0.25], *p *= 0.047; total problems: adjusted MD 0.80, 95% CI [0.40, 1.21], *p *< 0.001). Elevated scores on the conduct problems subscale and total problems were apparent across the whole sample during the 81-month data collection window (conduct problems: adjusted MD 0.12, 95% CI [0.02, 0.22], *p *= 0.020; total problems: 0.36, 95% CI [0.04, 0.68], *p *= 0.026). Findings were mixed, however, when drinking was classified by drinks per week. Sayal et al. (2007) found that alcohol intake of less than one drink per week during pregnancy was associated with poor total problems scores at 47 months (OR 1.48, 95% CI [1.05, 2.10]), 81 months (OR 1.62, 95% CI [1.10, 2.38]), and 93–108 months (OR 1.79, 95% CI [1.06, 3.00]) among girls. Furthermore, teacher-rated conduct score were also elevated in this exposure category for the whole sample at 93–108 months (OR 1.79, 95% CI [1.02, 1.94]). However, the association between PAE and total problems scores was not significant for one or more drinks per week or for male children.

An association between PAE and affect regulation was also found in infancy. Schoeps et al. (2018) found that four or more drinks per week was associated with children with higher total difficulties score at age 2 (Beta 0.13, *p *= 0.009). Using the Brief Infant-Toddler Social and Emotional Assessment (Briggs-Gowan et al., 2002) Halliday et al. (2017) found that early binge drinking with continued low level alcohol consumption over the duration of the pregnancy was associated with lower sensation avoidance scores in offspring at age 2. Maternal binge drinking during early pregnancy is also associated with a difficult temperament among six-month-old infants (Alvik et al., 2011).

One study found that light to moderate levels of drinking in early pregnancy was associated with a reduced likelihood of scores above the clinical cut-off on the CBCL in children aged between 2 and 14 years compared to unexposed children on the externalising (1 drink or less per week: OR 0.76, 95% CI [0.59, 0.99], *p *= 0.42; 2–6 drinks per week: OR 0.69, 95% CI [0.51, 0.93], *p *= 0.014; 7–10 drinks per week: OR 0.46, 95% CI [0.22, 1.00], *p *= 0.49), internalising (1 drink or less per week: OR 0.85, 95% CI [0.67, 1.07], *p *= 1.64; 2–6 drinks per week: OR 0.57, 95% CI [0.42, 0.76], *p *< 0.001; 7–10 drinks per week: OR 0.31, 95% CI [0.14, 0.69], *p *= 0.004), and total behaviour (1 drink or less per week: OR 0.82, 95% CI [0.63, 1.06], *p *= 1.33; 2–6 drink per week: OR 0.63, 95% CI [0.46, 0.76], *p *= 0.003; 7–10 drinks per week: OR 0.43, 95% CI [0.20, 0.88], *p *= 0.02) (Robinson et al., 2010). There was no significant effect on CBCL for heavy drinking (11 or more drinks per week) or for PAE in late pregnancy (i.e. 34 weeks).

### Adaptive behaviour, social skills, and communication

Two studies evaluated adaptive behaviour, social skills, and social communication (Halliday et al., 2017; Niclasen, Nybo Andersen, et al., 2014). Halliday et al. (2017) evaluated social competencies using the Brief Toddler Social Emotional Assessment (REF). The study found no significant association between PAE and social competencies. Niclasen, Nybo Andersen, et al. (2014) analysed the peer problems subscale of the SDQ. There was a significant increased risk of abnormal or borderline scores on the peer problems scale for children not exposed to alcohol compared to children to a cumulative 15–45 drinks over the pregnancy (Boys: RCM 1.21, 95% CI [1.03, 1.43]; Girls: RCM 1.40, 95% CI [1.15, 1.71]). The association between PAE and peer problems were not significant for any of the other alcohol exposure categories.

### Moderators

Across the 30 included studies, parental socio-economic characteristics was the only confounder to be consistently present. Socio-economic characteristics included one or multiple of the following: socioeconomic position, education, social class, house ownership, crowding, income, civil status, area-level deprivation, employment status, marital status, and family structure. Maternal age and maternal smoking during pregnancy were also commonly controlled for in the majority of analyses (maternal age; *n* = 27; maternal smoking: *n* = 26). Nineteen studies included parity or number of siblings as a key confounder in analyses. Other common variables include offspring gender (*n* = 15), maternal mental health during pregnancy (*n* = 14), and maternal physical health during pregnancy (*n* = 15). Other moderators, such as ethnicity, parenting behaviour, and other substance use were included in less than one-third of studies and can be found in Appendix 3.

Broadly, inclusion of moderators in the models reduced the significance of the models, for those that remained significant there was generally a small reduction in effect size, with some exceptions. Alvik et al. (2013) examined the effects of prenatal alcohol on behavioural symptoms, inclusion of moderators (maternal age, major lifetime depression, birth weight, H-SCL anxiety, H-SCL depression, education (mother and father), income mothers’ partner, and sex of child) produced a slight increase in the effect size for the greater than once a week binge exposure category. Sayal et al. (2007) examined the effect of prenatal alcohol on temperament, with the analyses split between boys and girls. For the models that were significant (girls) a slight increase in OR's was observed. Weile et al. (2020) observed an increase in hazard ratios after inclusion of confounding variables into the model examining associations between binge episodes and ADHD.

In most of the 30 studies all confounding variables discussed in the methods section were included in the model. D’Souza et al. (2019) measured a selection of prenatal variables which were treated as predictor variables and therefore not controlled for in analyses. These included maternal mental health (depression and stress), and maternal lifestyle factors (folate and smoking behaviour). In Falgreen Eriksen et al. (2012) birthweight and gestational age were considered to be mediators of the effects of alcohol exposure so were excluded from main analyses. Halliday et al. (2017) utilised a stepwise approach which resulted in the removal of moderator variables which were not significant in the model. This study conducted analyses on three outcome variables cognition (maternal age, maternal education, pre-pregnancy BMI, folate supplements in T1, folate supplements in T2/T3, household income), language (maternal age, ethnicity, maternal education, smoking in pregnancy, pre-pregnancy BMI, folate supplements in T2/T3), and motor (maternal education, parity, and household income) with the dropped variables shown in parentheses. Negrao et al. (2020) utilised a directed acyclic graph to identify confounding variables, as a result prematurity and intrauterine growth restriction was not identified as a confound or included in analyses. Niclasen, Andersen, et al. (2014) removed cumulated alcohol exposure from their analyses as it did not contribute significantly to the model. Finally, Sayal et al. (2007) listed child gender as a confound but conducted separate gender analysis and therefore did not control for it.

## Discussion

This review investigated the relationship between PAE and neurodevelopmental outcomes, while considering the effect of confounding variables. Overall, evidence of the effects of PAE on neurodevelopmental outcomes are mixed for most of the outcomes evaluated in this review. None of the studies reviewed found evidence of effects of PAE on executive function but there were varied effects for motor skills, cognition, language, academic achievement, attention, affect regulation, and adaptive behaviour and social skills. The presence or absence of adverse effects of PAE depended in part on the timing of exposure (e.g. early versus late pregnancy) and amount of exposure (e.g. binge versus daily drinking), although effects were not consistent across or within outcomes. Most studies included in this review were determined to be of good quality and unlikely to be biased by error based on the risk assessment completed on the NOS.

The most consistent effect found across studies was the impact of PAE on affect regulation. Seven out of eleven studies found adverse effects of PAE on affect regulation, particularly for heavy alcohol consumption or binge drinking during pregnancy (Alvik et al., 2011; Sayal et al., 2014; Skogerbo et al., 2013). Findings were more mixed with respect to low levels of alcohol consumption, with one study finding that low to moderate alcohol consumption (less than 10 standard units per week) was associated with a reduced risk in clinical behaviour problems.

A number of studies appeared to show low levels of alcohol use in pregnancy as protective against adverse neurodevelopmental outcomes (Kesmodel, Eriksen, et al., 2012; Niclasen, Nybo Andersen, et al., 2014; Robinson et al., 2010). This finding may reflect unexplored confounding variables in the non-drinking population. Previous research has consistently found that the non-drinking population has increased morbidity and mortality compared to occasional and light drinkers. The population of women that do not drink during pregnancy is also more likely to include populations experiencing increased hardship, such as refugees or those already at risk of adverse pregnancy outcomes. The population of low to moderate alcohol drinkers, in contrast, may be more affluent. A New Zealand birth cohort found that low levels of alcohol use throughout pregnancy was most common in older women (over 40), New Zealand European women, and women from socioeconomically *advantaged* backgrounds (household income over $150,000). Differences in socioeconomic status between women who engage in low to moderate alcohol use in pregnancy compared to women who engage in heavy alcohol use or abstain during pregnancy have also been reported in other cohorts (McCormack et al., 2018; Niclasen, Nybo Andersen, et al., 2014; Nykjaer et al., 2014; Zuccolo et al., 2013). For this reason, it is appropriate to distinguish between two groups of women who do not consume alcohol during pregnancy: women who abstain during pregnancy (but who consumed alcohol before pregnancy) and women who do not drink alcohol. This distinction has potential to control for socioeconomic differences between women who do not drink alcohol and women who drink low to moderately (Zuccolo et al., 2013).

Confounding variables could account for some of the variation between PAE and neurodevelopment outcomes. Long-term neurodevelopmental outcomes of PAE are susceptible to confounding variables in the home environment between the in-utero exposure and the outcome measurement. This study did not reveal any protective factors. All studies controlled for maternal baseline variables and other exposures in utero, however few of the studies included confounds relating to the environment after birth such as parenting styles, quality of the caregiving environment, and domestic relationships. Although the studies in this review controlled for known, measurable potential confounding factors, other unmeasured confounders in the postnatal environment that impact outcomes are possible, such as traumatic childhood events (Price et al., 2017), parents alcohol consumption (Huq et al., 2021) and child–parent relationship (Pinquart, 2017). For example, there is some evidence to suggest that PAE and exposure to traumatic events in childhood together result in more severe impairments in neurodevelopmental outcomes rather than just PAE alone (Price et al., 2017). Future research could further explore these postnatal confounding variables that may account for some of the variation between PAE and neurodevelopment outcomes.

The effects of PAE on neurodevelopmental outcomes may be different for male and female offspring. Specifically PAE was associated with negative effects on attention (Sayal et al., 2014) and affect regulation (Sayal et al., 2007; Sayal et al., 2014) for female offspring only, while one study found PAE had negative effect on affect regulation in males only (Niclasen, Andersen, et al., 2014). Perhaps the effects of PAE on neurodevelopmental outcomes, especially affect regulation, could be differential for males and females, due to different mechanism effecting how alcohol impacts development. However, due to only a few studies examining neurodevelopment outcomes for male and female offspring in this review it is difficult to draw conclusions on the effects of gender on the relationship. Therefore, future research should further investigate the potential effects of gender on PAE and neurodevelopmental outcomes, by examining results separately for each gender.

Most of the studies (*n* = 19) in this review assessed the neurodevelopmental outcomes in children under the age of 6. It could be that neurodevelopmental outcomes become more obvious, as children become older and their developmental demands change, and then impairments become easier to detect. Alternately, it could be that the way neurodevelopmental outcomes are measured is not as effective at detecting outcomes across all age groups. Regardless, people with FASD who access support services early have improved quality of life in part due to greater access to services and support (Domeij et al., 2018), therefore, it is essential not to delay diagnosis until the outcomes are more visible, but to be cautious in how FASD is diagnosed. For example, one study demonstrated eye movement technology was able to detect those with FASD from those without in children as young as 5 (Zhang et al., 2019).

Although confounding variables likely effect the relationship between PAE and neurodevelopmental outcomes, further variation could be due to how neurodevelopmental outcomes are conceptualised and measured. Each neurodevelopmental outcome is a complicated and multifaceted domain, so perhaps studies examining these outcomes only focus on part of the outcome rather than the whole domain. For instance, in the Canadian FASD guidelines (Cook et al., 2016) the neurodevelopmental outcome of adaptive behaviour, social skills or communication, covers people's ability to learn and adapt their behaviour to their environments, their ability to communication with others both verbally and non-verbally and their ability to maintain socially appropriate social relationships. To determine impairment in this domain, for a diagnosis with FASD, the recommended approach is to use informant interviews, direct observation and collection of information from records, to enable a clinician to make an informed judgement on the severity of impairment (Cook et al., 2016). The two studies that assessed this domain in this review, used questionnaires that assessed relationships with others (Halliday et al., 2017; Niclasen, Nybo Andersen, et al., 2014), with no studies looking at adaptive behaviour or communication. What is more, no studies in this review examined neurophysiological or memory outcomes, despite PAE likely resulting in impairments in these domains (Cook et al., 2016). Therefore, perhaps more research is needed for each neurodevelopment outcome to ensure the whole domain is being studied.

Most studies included in this review drew information from Western countries. Research into FASD in non-western countries is emerging, such as in South Africa (May et al., 2016) and children adopted from Russia and Ukraine (Colom et al., 2021), however this work is relatively new and focuses on the prevalence of FASD. Research of FASD in non-western countries difficult due to the lack of resources and FASD being largely unrecognised (Adnams, 2017), despite the estimated high prevalence of alcohol consumption during pregnancy (Popova et al., 2018). Two studies included in this review drew data from non-western countries, one from Brazil finding no association between PAE and cognition and inconsistent findings with motor skills (Negrao et al., 2020), another from South Africa found no association with motor skills or cognition (Donald et al., 2019), suggesting that research into PAE effects are in their infancy in non-western countries. Non-western counties are likely to experience different confounding variables due to different economic, social and cultural factors, such as the effects related to HIV or poverty in African countries (Adnams, 2017). Therefore, future research from non-western countries would be useful to provide further insight the effect of PAE on neurodevelopmental outcomes.

### Limitations

The results from this research should be considered in context of its limitations. Firstly, due to the nature of the research there are limitations in the types of studies that can be included. Observational research is unable to make strong causal inferences and as mentioned earlier is susceptible to problems of residual confounding. This review aimed to put an increased focus on confounding factors to gain a better understanding of the impact of environmental or maternal characteristics, other than PAE, that may negatively affect offspring outcomes. Although all of the included studies controlled for known confounding factors, very few controlled for other environmental factors, limiting possible analyses. Furthermore, large-scale cohort studies are often prone to attrition bias. Using the Newcastle Ottawa Scale (Penson et al., 2012), 63% of included studies were introduced to potential bias due to high levels of attrition.

Due to significant heterogeneity of the included studies a meta-analysis was not undertaken rather a narrative review was conducted. Heterogeneity was observed in many aspects of the included studies design. The definition of a standard drink, based on grams of alcohol, varied depending on country of origin. The classification of low, moderate, heavy, and binge drinking also varied between studies, with some studies using unique brackets of exposure. Across the eight neurodevelopmental outcomes assessed there were varying diagnostic tests or psychometrics utilised, as well as different means of reporting, for example, total scale scores versus an individual subscale score. Finally, the timeframes both for exposure assessments and outcome measurement varied greatly between studies, limiting comparability.

Consistently, across all of the included studies alcohol exposure was assessed by maternal self-report. Although primarily prospective methods were used (29 out of 30 studies) minimising retrospective recall bias, this form of measurement may still be prone to biases. Alcohol consumption is often susceptible to social desirability response bias, particularly in the case of the more stigmatised maternal consumption of alcohol (Davis et al., 2010). As a result, consumption may be under-reported. Furthermore, many of these studies conducted only one assessment of alcohol consumption. More regular assessment periods or use of different methodology may increase the quality of data produced and allow for more extensive analysis of pattern, frequency, and quantity of consumption across the pregnancy.

Finally, study inclusion was restricted to those published in the English language. However, previous research has indicated that exclusion of non-English language studies is unlikely to introduce systematic bias (Jüni et al., 2002; Moher et al., 2003; Morrison et al., 2012).

## Conclusions

Based on this comprehensive review of available large-scale cohort data, it is not possible to conclude a safe level of alcohol consumption during pregnancy. The longitudinal cohort studies produced mixed findings in most of the 10 neurodevelopmental domains considered in this review. Limitations outlined highlight the need to improve the quality and consistency in which PAE is studied, using methodology which may improve causal inferences. Finally, further exploration of residual confounding variables is vital, including characteristics of the environment after birth and via the separate classification of women who abstain from alcohol during pregnancy and those who do not drink at all.

## Supplementary Material

Supplemental Material

## Appendix 1. CINAHL search strategy.

**Table:**

#	Search terms	Results
S1	TI (((alcohol use) OR alcohol) AND (consum* OR expos* OR drink*)) OR AB (((alcohol use) OR alcohol) AND (consum* OR expos* OR drink*)) OR MW (((alcohol use) OR alcohol) AND (consum* OR expos* OR drink*))	52,344
S2	TI (matern* or pregnan* or f?etal or prenatal) OR AB (matern* or pregnan* or f?etal or prenatal) OR MW (matern* or pregnan* or f?etal or prenatal)	315,248
S3	TI (motor skills or neurophysiology or cognition or language or academic achievement or memory or attention or executive function or affect regulation or adaptive behavio?r or social skills or communication or fine motor or gross motor or IQ or intelligence or ((education* or school) and achievement) or inattention or emotional development or emotional regulation or behavio?r problems or developmental delay or neurodevelopment*) OR AB (motor skills or neurophysiology or cognition or language or academic achievement or memory or attention or executive function or affect regulation or adaptive behavio?r or social skills or communication or fine motor or gross motor or IQ or intelligence or ((education* or school) and achievement) or inattention or emotional development or emotional regulation or behavio?r problems or developmental delay or neurodevelopment*) OR MW (motor skills or neurophysiology or cognition or language or academic achievement or memory or attention or executive function or affect regulation or adaptive behavio?r or social skills or communication or fine motor or gross motor or IQ or intelligence or ((education* or school) and achievement) or inattention or emotional development or emotional regulation or behavio?r problems or developmental delay or neurodevelopment*)	565,786
S4	TI (prospective or longitudinal or follow-up or cohort) OR AB (prospective or longitudinal or follow-up or cohort) OR MW (prospective or longitudinal or follow-up or cohort)	818,896
S5	S1 AND S2	4085
S6	S3 AND S4 AND S5	173

## References

[CIT0001] Adnams, C. M. (2017). Fetal alcohol spectrum disorder in Africa. *Current Opinion in Psychiatry*, *30*(2), 108–112. 10.1097/YCO.0000000000000315 28125440

[CIT0002] Alati, R., Davey Smith, G., Lewis, S. J., Sayal, K., Draper, E. S., Golding, J., Fraser, R. & Gray, R. (2013). Effect of prenatal alcohol exposure on childhood academic outcomes: Contrasting maternal and paternal associations in the ALSPAC study. *PLoS One*, *8*(10), e74844. 10.1371/journal.pone.0074844 24130672 PMC3794033

[CIT0003] Alati, R., Macleod, J., Hickman, M., Sayal, K., May, M., Smith, G. D., & Lawlor, D. A. (2008). Intrauterine exposure to alcohol and tobacco use and childhood IQ: Findings from a parental-offspring comparison within the Avon longitudinal study of parents and children. *Pediatric Research*, *64*(6), 659–666. 10.1203/PDR.0b013e318187cc31 18670372

[CIT0004] Alvik, A., Aalen, O. O., & Lindemann, R. (2013). Early fetal binge alcohol exposure predicts high behavioral symptom scores in 5.5-year-old children. *Alcoholism: Clinical and Experimental Research*, *37*(11), 1954–1962. 10.1111/acer.12182 23888929

[CIT0005] Alvik, A., Torgersen, A. M., Aalen, O. O., & Lindemann, R. (2011). Binge alcohol exposure once a week in early pregnancy predicts temperament and sleeping problems in the infant. *Early Human Development*, *87*(12), 827–833. 10.1016/j.earlhumdev.2011.06.009 21757302

[CIT0006] Australian Government National Health Medical Research Council. (2009). *Australian guidelines to reduce health risks from drinking alcohol*. Commonwealth of Australia.

[CIT0007] Bayley, N. (2006). *Bayley scales of infant and toddler development, third edition*. Harcourt.

[CIT0008] Briggs-Gowan, M. J., Carter, A. S., Irwin, J. R., Wachtel, K., & Cicchetti, D. V. (2002). Brief Infant-Toddler Social and Emotional Assessment (BITSEA) mannual, version 2.0. 10.1093/jpepsy/jsh017 15096535

[CIT0009] Carson, G., Cox, L. V., Crane, J., Croteau, P., Graves, L., Kluka, S., Koren, G., Martel, M. J., Midmer, D., Nulman, I., & Poole, N. (2010). Alcohol use and pregnancy consensus clinical guidelines. *Journal of Obstetrics and Gynaecology Canada*, *32*(8, Supplement 3), S1–S2. 10.1016/S1701-2163(16)34633-3 21172102

[CIT0010] Carson, G., Cox, L. V., Crane, J., Croteau, P., Graves, L., Kluka, S., Koren, G., Martel, M. J., Midmer, D., Nulman, I., & Poole, N. (2017). No. 245-alcohol Use and pregnancy consensus clinical guidelines. *Journal of Obstetrics and Gynaecology Canada*, *39*(3), e220–e254. 10.1016/j.jogc.2017.06.005 28859770

[CIT0011] Colom, J., Segura-García, L., Bastons-Compta, A., Astals, M., Andreu-Fernandez, V., Barcons, N., Vidal, R., Ibar, A. I., Fumadó, V., Gómez, N., & Russiñol, A. (2021). Prevalence of Fetal Alcohol Spectrum Disorders (FASD) among children adopted from Eastern European countries: Russia and Ukraine. *International Journal of Environmental Research and Public Health*, *18*(1388), 1–12. 10.3390/ijerph18041388 PMC7913360 33546212

[CIT0012] Cook, J. L., Green, C. R., Lilley, C.M., Anderson, S. M., Baldwin, M. E., Chudley, A. E., Conry, J. L., LeBlanc, N., Loock, C. A., Lutke, J., & Mallon, B. F. (2016). Fetal alcohol spectrum disorder: A guidelines for diagnosis across the lifespan. *Canadian Medical Association Journal*, *188*(3), 191–197. 10.1503/cmaj.141593 26668194 PMC4754181

[CIT0013] Davis, C. G., Thake, J., & Vilhena, N. (2010). Social desirability biases in self-reported alcohol consumption and harms. *Addictive Behaviors*, *35*(4), 302–311. 10.1016/j.addbeh.2009.11.001 19932936

[CIT0014] Department of Health and Social Care. (2016). K Chief Medical Officers’ Low Risk Drinking Guidelines. Retrieved July 21, 2022, from https://assets.publishing.service.gov.uk/government/uploads/system/uploads/attachment_data/file/545937/UK_CMOs__report.pdf

[CIT0015] Domeij, H., Fahlstrom, G., Bertilsson, G., Hultcrantz, M., Munthe-Kaas, H., Gorhd, C. N., & Helgesson, G. (2018). Experiences of living with fetal alcohol spectrum disorders: A systematic review and synthesis of qualitative data. *Developmental Medicine & Child Neurology*, *60*(8), 741–752. 10.1111/dmcn.13696 29479676

[CIT0016] Donald, K. A., Wedderburn, C. J., Barnett, W., Nhapi, R. T., Rehman, A. M., Stadler, J. A., Hoffman, N., Koen, N., Zar, H. J., & Stein, D. J. (2019). Risk and protective factors for child development: An observational South African birth cohort. *PLOS Medicine*, *16*(9), 1–20. 10.1371/journal.pmed.1002920 PMC6764658 31560687

[CIT0017] D'Souza, S., Crawford, C. N., Buckley, J., Underwood, L., Peterson, E. R., Bird, A., Morton, S. M., & Waldie, K. E. (2019). Antenatal determinants of early childhood talking delay and behavioural difficulties. *Infant Behavior & Development*, *57*, 101388. 10.1016/j.infbeh.2019.101388 31634704

[CIT0018] Easey, K. E., Dyer, M. L., Timpson, N. J., & Munafo, M. R. (2019). Prenatal alcohol exposure and offspring mental health: A systematic review. *Drug and Alcohol Dependence*, *197*, 344–353. 10.1016/j.drugalcdep.2019.01.007 30827758 PMC6446223

[CIT0019] Faebo Larsen, R., Hvas Mortensen, L., Martinussen, T., & Nybo Andersen, A.-M. (2013). Determinants of developmental coordination disorder in 7-year-old children: A study of children in the Danish National Birth Cohort. *Developmental Medicine & Child Neurology*, *55*(11), 1016–1022. 10.1111/dmcn.12223 23909795

[CIT0020] Falgreen Eriksen, H. L., Mortensen, E. L., Kilburn, T., Underbjerg, M., Bertrand, J., Støvring, H., Wimberley, T., Grove, J., & Kesmodel, U. S. (2012). The effects of low to moderate prenatal alcohol exposure in early pregnancy on IQ in 5-year-old children. *BJOG: An International Journal of Obstetrics and Gynaecology*, *119*(10), 1191–1200. 10.1111/j.1471-0528.2012.03394.x 22712749 PMC4471997

[CIT0021] Floyd, R. L., & Sidhu, J. S. (2004). *Monitoring prenatal alcohol exposure.* Paper presented at the American Journal of Medical Genetics Part C: Seminars in Medical Genetics. 10.1002/ajmg.c.30010 15095466

[CIT0022] Ghazi Sherbaf, F., Aarabi, M. H., Hosein Yazdi, M., & Haghshomar, M. (2019). White matter microstructure in fetal alcohol spectrum disorders: A systematic review of diffusion tensor imaging studies. *Human Brain Mapping*, *40*(3), 1017–1036. 10.1002/hbm.24409 30289588 PMC6865781

[CIT0023] Gioia, G., Isquith, P., Guy, S., & Kenworth, L. (2000). Behaviour rating inventory of executive functions. *Child Neuropsychology*, *6*(3), 235–238. 10.1076/chin.6.3.235.3152 11419452

[CIT0024] Gupta, K. K., Gupta, V. K., & Shirasaka, T. (2016). An update on fetal alcohol syndrome—Pathogenesis. *Risks, and Treatment. Alcoholism: Clinical and Experimental Research*, *40*(8), 1594–1602. 10.1111/acer.13135 27375266

[CIT0025] Halliday, J. L., Muggli, E., Lewis, S., Elliott, E. J., Amor, D. J., O'Leary, C., Donath, S., Forster, D., Nagle, C., Craig, J. M., & Anderson, P. J. (2017). Alcohol consumption in a general antenatal population and child neurodevelopment at 2 years. *Journal of Epidemiology and Community Health*, *71*(10), 990–998. 10.1136/jech-2017-209165 28839077

[CIT0026] Harding, K., Flannigan, K., & McFarlane, A. A. (2019). *Policy action paper: Towards a standard definition of fetal alcohol spectrum disorder in Canada*. Canada: Canada Fetal Alcohol Spectrum Disorder Research Network.

[CIT0027] National Health and Medical Research Council. (2009). *Australian guidelines to reduce health risks from drinking alcohol*. National Health and Medical Research Council.

[CIT0028] Huq, T., Alexander, E. C., Manikam, L., Jokinen, T., Patil, P., Benjumea, D., Das, I., & Davidson, L. L. (2021). A systematic review of household and family alcohol use and childhood neurodevelopmental outcomes in low- and middle-income countries. *Child Psychiatry & Human Development*, *52*(6), 1194–1217. 10.1007/s10578-020-01112-3 33369706 PMC8528783

[CIT0029] Hutchins, T. (2013). MacArthur-Bates communicative development inventories, second edition. In F. R. Volkmar (Ed.), *Encyclopedia of autism spectrum disorders* (pp. 1773–1779). Springer New York.

[CIT0030] Hutchinson, D., Youssef, G. J., McCormack, C., Wilson, J., Allsop, S., Najman, J., Elliott, E., Burns, L., Jacobs, S., Honan, I., & Rossen, L. (2019). Prenatal alcohol exposure and infant gross motor development: A prospective cohort study. *BMC Pediatrics*, *19*(1), 149–149. 10.1186/s12887-019-1516-5 31088407 PMC6515673

[CIT0031] Jacobson, S. W., Jacobson, J. L., Sokol, R. J., Chiodo, L. M., & Corobana, R. (2004). Maternal age, alcohol abuse history, and quality of parenting as moderators of the effects of prenatal alcohol exposure on 7.5-year intellectual function. *Alcoholism: Clinical and Experimental Research*, *28*(11), 1732–1745. 10.1097/01.ALC.0000145691.81233.FA 15547461

[CIT0032] Jastak, S. (1984). *WRAT-R: Wide range achievement test: New and revised edition*. Wilmington, Del.: Jastak Associates, Inc.; Chicago, IL: Stoelting Co.

[CIT0033] Jüni, P., Holenstein, F., Sterne, J., Bartlett, C., & Egger, M. (2002). Direction and impact of language bias in meta-analyses of controlled trials: Empirical study. *International Journal of Epidemiology*, *31*(1), 115–123. 10.1093/ije/31.115 11914306

[CIT0034] Kesmodel, U. S., Bertrand, J., Stovring, H., Skarpness, B., Denny, C., & Mortensen, E. L. (2012). The effect of different alcohol drinking patterns in early to mid pregnancy on the child’s intelligence, attention, and executive function. *BJOG: An International Journal of Obstetrics and Gynaecology*, *119*(10), 1180–1190. 10.1111/j.1471-0528.2012.03393.x 22712700 PMC4435537

[CIT0035] Kesmodel, U. S., Eriksen, H. L., Underbjerg, M., Kilburn, T. R., Støvring, H., Wimberley, T., & Mortensen, E. L. (2012). The effect of alcohol binge drinking in early pregnancy on general intelligence in children. *BJOG*, *119*(10), 1222–1231. 10.1111/j.1471-0528.2012.03395.x 22712770

[CIT0036] Khoury, J. E., Milligan, K., & Girard, T. A. (2015). Executive functioning in children and adolescents prenatally exposed to alczohol: A meta-analytic review. *Neuropsychology Review*, *25*(2), 149–170. 10.1007/s11065-015-9289-6 26037669

[CIT0037] Kilburn, T. R., Eriksen, H. L. F., Underbjerg, M., Thorsen, P., Mortensen, E. L., Landrø, N. I., Bakketeig, L. S., Grove, J., Sværke, C., & Kesmodel, U. S. (2015). Low to moderate average alcohol consumption and binge drinking in early pregnancy: Effects on choice reaction time and information processing time in five-year-old children. *PLoS One*, *10*(9), e0138611. 10.1371/journal.pone.0138611 26382068 PMC4575046

[CIT0038] Lange, S., Probst, C., Gmel, G., Rehm, J., Burd, L., & Popova, S. (2017). Global prevalence of fetal alcohol spectrum disorder among children and youth: A systematic review and meta-analysis. *JAMA Pediatrics*, *171*(10), 948–956. 10.1001/jamapediatrics.2017.1919% 28828483 PMC5710622

[CIT0039] Mamluk, L., Edwards, H. B., Savović, J., Leach, V., Jones, T., Moore, T. H., Ijaz, S., Lewis, S. J., Donovan, J. L., Lawlor, D., & Smith, G. D. (2017). Low alcohol consumption and pregnancy and childhood outcomes: Time to change guidelines indicating apparently ‘safe’ levels of alcohol during pregnancy? A systematic review and meta-analyses. *BMJ Open*, *7*(7), e015410. 10.1136/bmjopen-2016-015410 PMC5642770 28775124

[CIT0040] Manly, T., Anderson, V., Nimmo-smith, I., Turner, A., Watson, P. Y., & Robertson, I. (2001). The differential assessment of children’s attention: The test of everyday attention for children (TEA-Ch), normative sample data and ADHD performance. *The Journal of Child Psychology and Psychiatry and Allied Disciplines*, *42*(8), 1065–1081. 10.1111/1469-7610.00806 11806689

[CIT0041] May, P. A., de Vries, M. M., Marais, A. S., Kalberg, W. O., Adnams, C. M., Hasken, J. M., Tabachnick, B., Robinson, L. K., Manning, M. A., Jones, K. L., & Hoyme, D. (2016). The continuum of fetal alcohol spectrum disorders in a community in South Africa: Prevalence and characteristics in a fifth sample. *Drug & Alcohol Dependence*, *168*, 274–286. 10.1016/j.drugalcdep.2016.09.025 27736681 PMC5086258

[CIT0042] McCormack, C., Hutchinson, D., Burns, L., Wilson, J., Elliott, E., Allsop, S., Najman, J., Jacobs, S., Rossen, L., Olsson, C., & Mattick, R. (2017). Prenatal alcohol consumption between conception and recognition of pregnancy. *Alcoholism, Clinical and Experimental Research*, *41*(2), 369–378. 10.1111/acer.13305 28116821

[CIT0043] McCormack, C., Hutchinson, D., Burns, L., Youssef, G., Wilson, J., Elliott, E., Allsop, S., Najman, J., Jacobs, S., Rossen, L., & Olsson, C. (2018). Maternal and partner prenatal alcohol use and infant cognitive development. *Drug & Alcohol Dependence*, *185*, 330–338. 10.1016/j.drugalcdep.2017.12.038 29499553

[CIT0044] Ministry of Health. (2020). Fetal alcohol spectrum disorder. 10 September 2018. Retrieved July 2, 2019, from https://www.health.govt.nz/our-work/diseases-and-conditions/fetal-alcohol-spectrum-disorder

[CIT0045] Moher, D., Liberati, A., Tetzlaff, J., Altman, D. G., & The, P. G. (2009). Preferred reporting items for systematic reviews and meta-analyses: The PRISMA statement. *PLOS Medicine*, *6*(7), e1000097. 10.1371/journal.pmed.1000097 19621072 PMC2707599

[CIT0046] Moher, D., Pham, B., Lawson, M. L., & Klassen, T. P. (2003). The inclusion of reports of randomised trials published in languages other than English in systematic reviews. *Health Technology Assessment*, *7*(41), 1–90. 10.3310/hta7410 14670218

[CIT0047] Morrison, A., Polisena, J., Husereau, D., Moulton, K., Clark, M., Fiander, M., … Rabb, D. (2012). The effect of English language restriction on systematic review-based meta-analyses: A systematic review of empirical studies. *International Journal of Technology Assessment in Health Care*, *28*(2), 138–144. 22559755 10.1017/S0266462312000086

[CIT0048] Murphy, D. J., Mullally, A., Cleary, B. J., Fahey, T., & Barry, J. (2013). Behavioural change in relation to alcohol exposure in early pregnancy and impact on perinatal outcomes-a prospective cohort study. *BMC Pregnancy and Childbirth*, *13*(1), 1–8. 10.1186/1471-2393-13-8 23324650 PMC3549282

[CIT0049] Negrão, M. E. A., Rocha, P. R. H., Saraiva, M. C. P., Barbieri, M. A., Simões, V. M. F., Batista, R. F. L., Ferraro, A. A., & Bettiol, H. (2020). Association between tobacco and/or alcohol consumption during pregnancy and infant development: Brisa cohort. *Brazilian Journal of Medical and Biological Research*, *54*(1), 1–9. 10.1590/1414-431×202010252 PMC7780373 33338100

[CIT0050] Niclasen, J., Andersen, A.-M., Strandberg-Larsen, K., & Teasdale, T. (2014). Is alcohol binge drinking in early and late pregnancy associated with behavioural and emotional development at age 7 years? *European Child & Adolescent Psychiatry*, *23*(12), 1175–1180. 10.1007/s00787-013-0511-x 24390718

[CIT0051] Niclasen, J., Nybo Andersen, A., Teasdale, T., & Strandberg-Larsen, K. (2014). Prenatal exposure to alcohol, and gender differences on child mental health at age seven year. *Journal of Epidemiology and Community Health*, *68*(3), 224–232. 10.1136/jech-2013-202956 24218073

[CIT0052] Nykjaer, C., Alwan, N. A., Greenwood, D. C., Simpson, N. A., Hay, A. W., White, K. L., & Cade, J. E. (2014). Maternal alcohol intake prior to and during pregnancy and risk of adverse birth outcomes: Evidence from a British cohort. *Journal of Epidemiology and Community Health*, *68*(6), 542–549. 10.1136/jech-2013-202934 24616351 PMC4033207

[CIT0053] O’Callaghan, F. V., O’Callaghan, M., Najman, J. M., Williams, G. M., & Bor, W. (2007). Prenatal alcohol exposure and attention, learning and intellectual ability at 14 years: A prospective longitudinal study. *Early Human Development*, *83*(2), 115–123. 10.1016/j.earlhumdev.2006.05.011 16842939

[CIT0054] Penson, D., Krishnaswami, S., Jules, A., Seroogy, J., & McPheeters, M. (2012). *Newcastle-Ottawa quality assessment form for cohort studies*. Rockville, MD: Agency for Healthcare Research and Quality (US). 23326894

[CIT0055] Pinquart, M. (2017). Associations of parenting dimensions and styles with externalizing problems of children and adolescents: An updated meta-analysis. *Developmental Psychology*, *53*(5), 873–932. 10.1037/dev0000295 28459276

[CIT0056] Popova, S., Lange, S., Probst, C., Gmel, G., & Rehm, J. (2018). Global prevalence of alcohol use and binge drinking during pregnancy, and fetal alcohol spectrum disorder. Biochemistry and Cell Biology, *96*(2), 237–240. 10.1139/bcb-2017-0077%M28834683 28834683

[CIT0057] Price, A., Cook, P. A., Norgate, S., & Mukherjee, R. (2017). Prenatal alcohol exposure and traumatic childhood experiences: A systematic review. *Neuroscience and Biobehavioral Reviews*, *80*, 89–98. 10.1016/j.neubiorev.2017.06.018 28552459

[CIT0058] Raven, J., Raven, J. C., & Court, J. H. (1998). *Manual for Raven’s progressive matrices and vocabulary scales*. Oxford Psychologists Press.

[CIT0059] Robinson, M., Oddy, W. H., McLean, N. J., Jacoby, P., Pennell, C. E., De Klerk, N. H., Zubrick, S. R., Stanley, F. J., & Newnham, J. P. (2010). Low–moderate prenatal alcohol exposure and risk to child behavioural development: A prospective cohort study. *BJOG: An International Journal of Obstetrics & Gynaecology*, *117*(9), 1139–1152. 10.1111/j.1471-0528.2010.02596.x 20528867

[CIT0060] Rodriguez, A., Olsen, J., Kotimaa, A. J., Kaakinen, M., Moilanen, I., Henriksen, T. B., … Järvelin, M. R. (2009). Is prenatal alcohol exposure related to inattention and hyperactivity symptoms in children? Disentangling the effects of social adversity. *Journal of Child Psychology & Psychiatry*, *50*(9), 1073–1083. 10.1111/j.1469-7610.2009.02071.x 19298478

[CIT0061] Rossen, F., Newcombe, D., Parag, V., Underwood, L., Marsh, S., Berry, S., Grant, C., Morton, S., & Bullen, C. (2018). Alcohol consumption in New Zealand women before and during pregnancy: Findings from the growing up in New Zealand study. *Alcohol*, *131*(1479), 24–34. 30048430

[CIT0062] San Martin Porter, M., Maravilla, J. C., Betts, K. S., & Alati, R. (2019). Low-moderate prenatal alcohol exposure and offspring attention-deficit hyperactivity disorder (ADHD): Systematic review and meta-analysis. *Archives of Gynaecology and Obstetrics*, *300*(2), 269–277. 10.1007/s00404-019-05204-x 31161393

[CIT0063] Sayal, K., Draper, E. S., Fraser, R., Barrow, M., Davey Smith, G., & Gray, R. (2013). Light drinking in pregnancy and mid-childhood mental health and learning outcomes. *Archives of Disease in Childhood*, *98*(2), 107–111. 10.1136/archdischild-2012-302436 23322857 PMC3551199

[CIT0064] Sayal, K., Heron, J., Draper, E., Alati, R., Lewis, S. J., Fraser, R., Barrow, M., Golding, J., Emond, A., Davey Smith, G., & Gray, R. (2014). Prenatal exposure to binge pattern of alcohol consumption: Mental health and learning outcomes at age 11. *European Child & Adolescent Psychiatry*, *23*(10), 891–899. 10.1007/s00787-014-0599-7 25209690 PMC4186965

[CIT0065] Sayal, K., Heron, J., Golding, J., Alati, R., Smith, G. D., Gray, R., & Emond, A. (2009). Binge pattern of alcohol consumption during pregnancy and childhood mental health outcomes: Longitudinal population-based study. *Pediatrics*, *123*(2), e289–e296. 10.1542/peds.2008-1861 19171582 PMC6485410

[CIT0066] Sayal, K., Heron, J., Golding, J., & Emond, A. (2007). Prenatal alcohol exposure and gender differences in childhood mental health problems: A longitudinal population-based study. *Pediatrics*, *119*(2), e426–e434. 10.1542/peds.2006-1840 17272604

[CIT0067] Schoeps, A., Peterson, E. R., Mia, Y., Waldie, K. E., Underwood, L., D’Souza, S., & Morton, S. M. B. (2018). Prenatal alcohol consumption and infant and child behavior: Evidence from the growing up in New Zealand cohort. *Early Human Development*, *123*, 22–29. 10.1016/j.earlhumdev.2018.06.011 30036725

[CIT0068] Skogerbo, A., Kesmodel, U. S., Denny, C. H., Kjaersgaard, M. I. S., Wimberley, T., Landro, N. I., & Mortensen, E. L. (2013). The effects of low to moderate alcohol consumption and binge drinking in early pregnancy on behaviour in 5-year-old children: A prospective cohort study on 1628 children. *BJOG: An International Journal of Obstetrics and Gynaecology*, *120*(9), 1042–1050. 10.1111/1471-0528.12208 23837773 PMC4447188

[CIT0069] Skogerbo, A., Kesmodel, U. S., Wimberley, T., Stovring, H., Bertrand, J., Landro, N. I., & Mortensen, E. L. (2012). The effects of low to moderate alcohol consumption and binge drinking in early pregnancy on executive function in 5-year-old children. *BJOG: An International Journal of Obstetrics and Gynaecology*, *119*(10), 1201–1210. 10.1111/j.1471-0528.2012.03397.x 22712874 PMC4469190

[CIT0070] Skorka, K., McBryde, C., Copley, J., Meredith, P. J., & Reid, N. (2020). Experiences of children with fetal alcohol spectrum disorder and their families: A critical review. Alcoholism: Clinical and Experimental Research, *44*(6), 1175–1188. 10.1111/acer.14335 32282931

[CIT0071] Streissguth, A. P., Bookstein, F. L., Barr, H. M., Sampson, P. D., O’malley, K., & Young, J. K. (2004). Risk factors for adverse life outcomes in fetal alcohol syndrome and fetal alcohol effects. *Journal of Developmental & Behavioral Pediatrics*, *25*(4), 228–238. 10.1097/00004703-200408000-00002 15308923

[CIT0072] Sundermann, A. C., Zhao, S., Young, C. L., Lam, L., Jones, S. H., Velez Edwards, D. R., & Hartmann, K. E. (2019). Alcohol use in pregnancy and miscarriage: A systematic review and meta-analysis. *Alcoholism: Clinical and Experimental Research*, *43*(8), 1606–1616. 10.1111/acer.14124 31194258 PMC6677630

[CIT0073] Falgreen Eriksen, H. L., Mortensen, E. L., Kilburn, T., Underbjerg, M., Bertrand, J., Støvring, H., Wimberley, T., Grove, J., & Kesmodel, U. S. (2012). The effects of low to moderate alcohol consumption and binge drinking in early pregnancy on selective and sustained attention in 5-year-old children. *BJOG: An International Journal of Obstetrics and Gynaecology*, *119*(10), 1211–1221. 10.1111/j.1471-0528.2012.03396.x 22712829

[CIT0074] Wechsler, D. (2003). *Wechsler intelligence scale for children*. The Psychological Corporation.

[CIT0075] Wechsler, D. (2012). *Wechsler preschool and primary scale of intelligence—fourth edition*. The Pyschological Corporation. 10.1037/spq0000038 24188289

[CIT0076] Weile, L. K. K., Wu, C., Hegaard, H. K., Kesmodel, U. S., Henriksen, T. B., & Nohr, E. A. (2020). Alcohol intake in early pregnancy and risk of attention-deficit/hyperactivity disorder in children Up to 19 years of age: A cohort study. *Alcoholism: Clinical & Experimental Research*, *44*(1), 168–177. 10.1111/acer.14243 31742728

[CIT0077] Wilson, B., Crawford, S., Green, D., Roberts, G., Aylott, A., & Kaplan, B. (2009). Psychometric properties of the revised developmental coordination disorder questionnaire. *Physical & Occupational Therapy in Pediatrics*, *29*(2), 182–202. 10.1080/01942630902784761 19401931

[CIT0078] Zhang, C., Paolozza, A., Tseng, P., Reynolds, J. N., Munoz, D. P., & Itti, L. (2019). Detection of children/youth with fetal alcohol spectrum disorder through eye movement, psychometric, and neuroimaging data. *Frontiers in Neurology*, *10*(90), 80–80. 10.3389/fneur.2019.00080 30833926 PMC6388649

[CIT0079] Zuccolo, L., Lewis, S. J., Davey Smith, G., Sayal, K., Draper, E. S., Fraser, R., Barrow, M., Alati, R., Ring, S., Macleod, J., & Golding, J. (2013). Prenatal alcohol exposure and offspring cognition and school performance. A ‘Mendelian randomization’ natural experiment. *International Journal of Epidemiology*, *42*(5), 1358–1370. 10.1093/ije/dyt172 24065783 PMC3807618

