# Supplementary material for: Impact of prenatal alcohol exposure on neurodevelopmental outcomes: a systematic review
Source: Health Psychol Behav Med. 2022 Oct 6;10(1):973–1002. doi: 10.1080/21642850.2022.2129653 (PMC9553152; doi:10.1080/21642850.2022.2129653)
Supplement: Supplemental Material [file RHPB_A_2129653_SM4745.docx]

Appendix 1: CINAHL Search Strategy

| # | Search Terms | Results |
| --- | --- | --- |
| S1 | TI ( ((alcohol use) OR alcohol) AND (consum* OR expos* OR drink*) ) OR AB ( ((alcohol use) OR alcohol) AND (consum* OR expos* OR drink*) ) OR MW ( ((alcohol use) OR alcohol) AND (consum* OR expos* OR drink*) ) | 52,344 |
| S2 | TI ( matern* or pregnan* or f?etal or prenatal ) OR AB ( matern* or pregnan* or f?etal or prenatal ) OR MW ( matern* or pregnan* or f?etal or prenatal ) | 315,248 |
| S3 | TI ( motor skills or neurophysiology or cognition or language or academic achievement or memory or attention or executive function or affect regulation or adaptive behavio?r or social skills or communication or fine motor or gross motor or IQ or intelligence or ((education* or school) and achievement) or inattention or emotional development or emotional regulation or behavio?r problems or developmental delay or neurodevelopment* ) OR AB ( motor skills or neurophysiology or cognition or language or academic achievement or memory or attention or executive function or affect regulation or adaptive behavio?r or social skills or communication or fine motor or gross motor or IQ or intelligence or ((education* or school) and achievement) or inattention or emotional development or emotional regulation or behavio?r problems or developmental delay or neurodevelopment* ) OR MW ( motor skills or neurophysiology or cognition or language or academic achievement or memory or attention or executive function or affect regulation or adaptive behavio?r or social skills or communication or fine motor or gross motor or IQ or intelligence or ((education* or school) and achievement) or inattention or emotional development or emotional regulation or behavio?r problems or developmental delay or neurodevelopment* ) | 565,786 |
| S4 | TI ( prospective or longitudinal or follow-up or cohort ) OR AB ( prospective or longitudinal or follow-up or cohort ) OR MW ( prospective or longitudinal or follow-up or cohort ) | 818,896 |
| S5 | S1 AND S2 | 4,085 |
| S6 | S3 AND S4 AND S5 | 173 |

Appendix 2: Quality of Included Studies

## Supplementary Table S2. Risk of bias assessment scores based on NOS scale of cohort, longitudinal and cross-sectional studies

| **Study** | Exposed cohort | Non-exposed cohort | | Exposure | Outcome Timing | Comparability | | Assessment | Length of follow-up | | Adequacy of follow-up cohorts | | **Total** |
| --- | --- | --- | --- | --- | --- | --- | --- | --- | --- | --- | --- | --- | --- |
| Alati et al. (2013) | 1 | 1 | | 1 | 1 | 2 | | 1 | 1 | | 0 | | **8** |
| Alati et al. (2008) | 1 | 1 | | 1 | 1 | 2 | | 1 | 1 | | 0 | | **8** |
| Alvik et al. (2013) | 1 | 1 | | 1 | 1 | 2 | | 1 | 1 | | 0 | | **8** |
| Alvik et al. (2011) | 1 | 1 | | 1 | 1 | 2 | | 0 | 0 | | 0 | | **6** |
| D'Souza et al. (2019) | 1 | 1 | | 1 | 1 | 1 | | 0 | 1 | | 0 | | **6** |
| Donald et al. (2019) | 1 | 1 | | 1 | 1 | 2 | | 1 | 1 | | 0 | | **8** |
| Faebo Larsen et al. (2013) | 1 | 1 | | 1 | 1 | 2 | | 0 | 1 | | 0 | | **7** |
| Falgreen Eriksen et al. (2012) | 0 | 1 | | 1 | 1 | 2 | | 1 | 1 | | 1 | | **8** |
| Halliday et al. (2017) | 1 | 1 | | 1 | 1 | 2 | | 1 | 1 | | 0 | | **8** |
| Hutchinson et al. (2019) | 0 | 1 | | 1 | 1 | 2 | | 1 | 0 | | 0 | | **6** |
| Kesmodel et al. (2012a) | 0 | 1 | | 1 | 1 | 2 | | 1 | 1 | | 1 | | **8** |
| Kesmodel et al. (2012b) | 0 | 1 | | 1 | 1 | 2 | | 1 | 1 | | 1 | | **8** |
| McCormack et al. (2018) | 0 | 1 | | 1 | 1 | 2 | | 1 | 0 | | 0 | | **6** |
| Negrao et al. (2020) | 0 | 1 | | 0 | 1 | 2 | | 1 | 0 | | 0 | | **5** |
| Niclasen et al. (2014a) | 1 | 1 | | 1 | 1 | 2 | | 0 | 1 | | 1 | | **8** |
| Niclasen et al. (2014b) | 1 | 1 | | 1 | 1 | 2 | | 0 | 1 | | 0 | | **7** |
| O'Callaghan et al. (2007) | 0 | 1 | | 1 | 1 | 2 | | 1 | 1 | | 0 | | **7** |
| Rodriguez et al. (2009) | 1 | 1 | | 1 | 1 | 2 | | 1 | 1 | | 0 | | **8** |
| Sayal et al. (2013) | 1 | 1 | | 1 | 1 | 2 | | 1 | 1 | | 0 | | **8** |
| Sayal et al. (2014) | 1 | 1 | | 1 | 1 | 2 | | 1 | 1 | | 0 | | **8** |
| Sayal et al. (2009) | 1 | 1 | | 1 | 1 | 2 | | 1 | 1 | | 0 | | **8** |
| Sayal et al. (2007) | 1 | 1 | | 1 | 1 | 2 | | 1 | 1 | | 0 | | **8** |
| Schoeps et al. (2018) | 1 | 1 | 1 | | 1 | | 2 | 0 | | 1 | | 1 | **8** |
| Skogerbo et al. (2013) | 0 | 1 | 1 | | 1 | | 2 | 1 | | 1 | | 1 | **8** |
| Skogerbo et al. (2012) | 0 | 1 | 1 | | 1 | | 2 | 1 | | 1 | | 1 | **8** |
| Underbjerg et al. (2012) | 0 | 1 | 1 | | 1 | | 2 | 1 | | 1 | | 1 | **8** |
| Weile et al. (2020) | 1 | 1 | 1 | | 1 | | 2 | 1 | | 1 | | 1 | **9** |
| Zuccolo et al. (2013) | 1 | 1 | 1 | | 1 | | 2 | 1 | | 1 | | 1 | **9** |
| Kilburn et al. (2015) | 0 | 1 | 1 | | 1 | | 2 | 1 | | 1 | | 1 | **8** |
| Robinson et al. (2010) | 0 | 1 | 1 | | 1 | | 2 | 0 | | 1 | | 0 | **6** |

Appendix 3: Confounders

**Supplementary Table S3. Confounders**

| Article Number | 1 | 2 | 3 | 4 | 5 | 6 | 7 | 8 | 9 | 10 | 11 | 12 | 13 | 14 | 15 | 16 | 17 | 18 | 19 | 20 | 21 | 22 | 23 | 24 | 25 | 26 | 27 | 28 | 29 | 30 | Total |
| --- | --- | --- | --- | --- | --- | --- | --- | --- | --- | --- | --- | --- | --- | --- | --- | --- | --- | --- | --- | --- | --- | --- | --- | --- | --- | --- | --- | --- | --- | --- | --- |
| **Confounder** |  |  |  |  |  |  |  |  |  |  |  |  |  |  |  |  |  |  |  |  |  |  |  |  |  |  |  |  |  |  |  |
| Offspring gender | x | x | x | x | x | - | x | x | x | - | x | x | - | - | - | - | - | - | - | - | - | - | x | x | x | x | - | - | x | - | 15 |
| Offspring age |  | - | - | - | x | - | - | x | - | - | x | x | - | - | - | - | - | - | - | - | - | - | - | x | x | x | - | - | x | x | 9 |
| Offspring comorbid externalising disorders | - | - | - | - | - | - | - | - | - | - | - | - | - | - | - | - | - | - | - | - | - | - | - | - | - | x | - | - | - | - | 1 |
| Parity and/or number of siblings | x | x | - | - | x | - | - | x | x | x | x | x | x | - | - | - | - | - | x | x | x | x | - | x | x | x | x | x | x | - | 19 |
| Maternal ethnicity | x | x | - | - | x | - | - | - | - | x | - | - | x | - | - | - | - | - | - | - | x | x | x | - | - | - | - | - | - | - | 8 |
| Maternal age at offspring birth | x | - | x | x | x | - | x | x | x | x | x | x | x | x | x | - | x | x | x | x | x | x | x | x | x | x | x | x | x | x | 27 |
| Parental socio-economic characteristics (social class, education, income, marital status) | x | x | x | x | x | x | x | x | x | x | x | x | x | x | x | x | x | x | x | x | x | x | x | x | x | x | x | x | x | x | 30 |
| Parenting behaviour and/or home environment | - | - | - | - | - | - | - | x | x | - | x | x | - | - | - | - | - | - | - | - | - | - | - | x | x | x | - | - | x | - | 8 |
| Other parental psychopathology (including externalising disorders) and substance use disorders | - | - | x | - | - | - | - | - | x | - | - | - | - | - | x | x | - | - | - | - | - | x | - | - | - | x | - | x | - | - | 7 |
| Maternal mental health during pregnancy | - | - | x | x | - | x | - | - | x | x | - | - | x | x | - | x | - | - | x | x | x | x | x | - | - | - | - | - | - | x | 14 |
| Maternal other substance use during pregnancy | - | - | - | - | - | - | - | - | - | x | - | - | x | - | - | - | - | - | x | x | x | x | - | - | - | - | - | - | - | - | 6 |
| Maternal smoking during pregnancy | x | x | - | x | - | - | x | x | x | x | x | x | x | - | x | x | x | x | x | x | x | x | x | x | x | x | x | x | x | x | 26 |
| Partner’s or household member substance use during pregnancy | - | - | - | - | - | - | - | - | - | - | - | - | - | - | - | - | - | - | - | - | - | - | - | - | - | - | - | - | - | - | 0 |
| Paternal drinking during pregnancy | x | x | - | - | - | - | - | - | - | - | - | - | - | - | - | - | - | - | - | - | - | - | - | - | - | - | - | - | - | - | 2 |
| Parental postnatal drinking | - | - | - | - | - | - | - | - | - | - | - | - | - | - | - | - | - | - | - | - | - | - | - | - | - | x | x | - | - | - | 2 |
| Paternal smoking during pregnancy | x | x | - | - | - | - | - | - | - | - | - | - | - | - | - | x | - | - | - | - | - | - | - | - | - | - | - | - | - | - | 3 |
| Parental postnatal smoking | - | - | - | - | - | - | - | x | - | - | x | x | - | - | - | - | - | - | - | - | - | - | - | x | x | x | - | - | x | - | 7 |
| Planned/unplanned pregnancy | - | - | - | x | x | - | - | - | - | - | - | - | - | - | - | - | - | - | - | - | - | - | x | - | - | - | - | - | - | - | 3 |
| Maternal physical health in pregnancy (e.g. anaemia, BMI, diet, folate supplements) | - | - | - | - | - | x | - | x | x | x | x | x | x | - | - | x | x | - | - | - | - | - | - | x | x | x | x | x | x | - | 15 |
| Breastfeeding | - | - | - | - | - | - | - | - | x | - | - | - | - | - | - | - | - | - | - | - | - | - | - | - | - | - | - | - | - | - | 1 |
| Perinatal factors (birth weight, gestational age, birth complications) | - | - | x | x | x | x | x | - | - | x | - | - | x | - | - | - | - | x | x | x | x | x | x | x | - | - | - | - | - | - | 14 |
| Offspring health status | - | - | - | - | - | - | - | x | - | - | x | x | - | - | - | - | - | - | - | - | - | - | - | x | x | x | - | - | x | - | 7 |
